# Cross-cultural adaptation, reliability and validity of the Fremantle Knee Awareness Questionnaire in Italian subjects with painful knee osteoarthritis

**DOI:** 10.1186/s12955-021-01754-4

**Published:** 2021-04-07

**Authors:** Marco Monticone, Cristiano Sconza, Igor Portoghese, Tomohiko Nishigami, Benedict M. Wand, Gregorio Sorrentino, Giulia Lemorini, Stefano Respizzi, Andrea Giordano, Franco Franchignoni

**Affiliations:** 1 Department of Medical Sciences and Public Health, University of Cagliari, Cagliari, Italy; 2 Neurorehabilitation Unit, Department of Neuroscience and Rehabilitation, G. Brotzu Hospital, Cagliari, Italy; 3 Department of Physical and Rehabilitation Medicine, Humanitas Clinical and Research Centre IRCSS, Rozzano, Milan, Italy; 4 Department of Medical Sciences and Public Health, University of Cagliari, Cittadella Universitaria, Strada Statale, 554 - Monserrato, Cagliari, Italy; 5 Faculty of Health and Welfare, Prefectural University of Hiroshima, Mihara, Hiroshima, Japan; 6 The School of Physiotherapy, The University of Notre Dame Australia, Fremantle, WA Australia; 7 Physical Medicine and Rehabilitation School, University of Milano-Bicocca, Milan, Italy; 8 Physical Medicine and Rehabilitation School, University of Milan, Milan, Italy; 9 ICS Maugeri, IRCCS, Bioengineering Unit, Institute of Veruno, Veruno, NO Italy; 10 ICS Maugeri, IRCCS, Physical Medicine and Rehabilitation Unit, Institute of Tradate, Tradate, VA Italy

**Keywords:** Knee osteoarthritis, Rehabilitation, Outcome measure, Cross-cultural adaptation, Validity, Reliability

## Abstract

**Background and aim:**

Growing attention is being given to utilising physical function measures to better understand and manage knee osteoarthritis (OA). The Fremantle Knee Awareness Questionnaire (FreKAQ), a self-reported measure of body-perception specific to the knee, has never been validated in Italian patients. The aims of this study were to culturally adapt and validate the Italian version of the FreKAQ (FreKAQ-I), to allow for its use with Italian-speaking patients with painful knee OA.

**Methods:**

The FreKAQ-I was developed by means of forward–backward translation, a final review by an expert committee and a test of the pre-final version to evaluate its comprehensibility. The psychometric testing included: internal structural validity by Rasch analysis; construct validity by assessing hypotheses of FreKAQ correlations with the knee injury and osteoarthritis outcome score (KOOS), a pain intensity numerical rating scale (PI-NRS), the pain catastrophising scale (PCS), and the Hospital anxiety and depression score (HADS) (Pearson’s correlations); known-group validity by evaluating the ability of FreKAQ scores to discriminate between two groups of participants with different clinical profiles (Mann–Whitney U test); reliability by internal consistency (Cronbach’s alpha) and test–retest reliability (intraclass correlation coefficient, ICC_2.1_); and measurement error by calculating the minimum detectable change (MDC).

**Results:**

It took one month to develop a consensus-based version of the FreKAQ-I. The questionnaire was administered to 102 subjects with painful knee OA and was well accepted. Internal structural validity confirmed the substantial unidimensionality of the FreKAQ-I: variance explained was 53.3%, the unexplained variance in the first contrast showed an eigenvalue of 1.8, and no local dependence was detected. Construct validity was good as all of the hypotheses were met; correlations: KOOS (rho = 0.38–0.51), PI-NRS (rho = 0.35–0.37), PCS (rho = 0.47) and HADS (Anxiety rho = 0.36; Depression rho = 0.43). Regarding known-groups validity, FreKAQ scores were significantly different between groups of participants demonstrating high and low levels of pain intensity, pain catastrophising, anxiety, depression and the four KOOS subscales (*p* ≤ 0.004). Internal consistency was acceptable (α = 0.74) and test–retest reliability was excellent (ICC = 0.92, CI 0.87–0.94). The MDC_95_ was 5.22 scale points.

**Conclusion:**

The FreKAQ-I is unidimensional, reliable and valid in Italian patients with painful knee OA. Its use is recommended for clinical and research purposes.

**Supplementary Information:**

The online version contains supplementary material available at 10.1186/s12955-021-01754-4.

## Background

Knee osteoarthritis (OA) occurs due to a broad variety of factors, including genetic, metabolic, biomechanical, or post-traumatic causes [1]. It usually develops over a 10- to 15-year period due to articular cartilage damage, bony osteophyte formation and sclerosis of the subchondral bone [2]. Patients commonly report local pain after prolonged rest, stiffness, reduced range of motion, muscle weakness, swelling, and locking [3]. These clinical manifestations lead to difficulty in functional activities such as prolonged sitting, walking and climbing stairs, and an overall reduced quality of life [4].

People with persistent pain secondary to knee OA also exhibit disruption of some of the mechanisms thought to underpin body-perception, including impairments in limb laterality recognition [5] as well as reduced proprioceptive [6, 7] and tactile [8] acuity. Based on this premise, a new scale, derived from the Fremantle Back Awareness Questionnaire [9], namely the Fremantle Knee Awareness Questionnaire (FreKAQ), was recently introduced to directly measure altered body-perception specific to the knee. The developers demonstrated its unidimensionality, and that its score can provide a measure of perceptual impairment for patients suffering from knee OA [10]. Further, the FreKAQ showed acceptable internal consistency, test–retest reliability, and construct validity [10]. Indeed, it was correlated with pain intensity, disability, pain related catastrophising, kinesiophobia and anxiety [10].

Consequently, the FreKAQ attracted our attention as a useful means of assessing symptoms of impaired body-perception in subjects suffering from chronic knee OA, in order to comprehensively plan conservative treatments. However, there is potential for considerable variability in the performance of a patient-reported outcome measure when cross-culturally adapted and used in a different country from where it was originally developed [11]. Established methodological standards are advocated when validation studies are conducted, and these studies contribute to increasing the quality of an outcome measure, allowing for more robust comparison of results across different countries [12].

As an Italian version of the FreKAQ has not been cross-culturally adapted and psychometrically analysed, Italian researchers and clinicians are limited from using this instrument. Therefore, the aims of this study were to develop an adapted Italian version of the questionnaire (FreKAQ-I), evaluate—in individuals with painful knee osteoarthritis- its reliability by assessing internal consistency and test–retest reliability, measurement error by assessing minimum detectable change, as well as construct validity by Rasch analysis and testing a priori hypotheses regarding associations between FreKAQ-I and measures of knee pain related disability, pain intensity, pain related catastrophising and mood.

## Methods

This cross-sectional study was approved by our Local Ethical Committee on the 24/10/2019 (no. 2386), and conducted in accordance with ethical and humane principles of research outlined in the Declaration of Helsinki.

### Participants

The study involved people attending the Rehabilitation Unit of a Research Hospital in Milan (Italy), between November 2019 and May 2020. The inclusion criteria were: primary knee OA ruled in by X-ray examination [13]; stable knee pain of at least 3 months duration; an age of > 18 years; and fluency in Italian (i.e. the ability to read and write). Exclusion criteria were: other causes of knee pain (e.g. previous lower limb surgery, infection, fracture, osteonecrosis or malignancy); systemic illness; recent myocardial infarctions or cerebrovascular events, ruled in by imaging (radiographs and, in doubtful cases, Computed Tomography or Magnetic Resonance Imaging) and/or case history; mental health/psychiatric problems (Mini-Mental State Examination scale of < 24); and unwillingness or inability to provide informed consent.

### Procedure

Outpatients visiting the centre during the study period were evaluated by two physiatrists, coordinated by the principal investigator. Those satisfying the inclusion criteria were invited to sign a written informed consent form. Once the patients had given their approval to participate in the study, their demographic and clinical characteristics were recorded by a research assistant and participants completed the questionnaires listed below. All participants were asked to complete the FreKAQ-I a second time 8 ± 2 (mean ± standard deviation) days after their initial appointment.

### Measures

*FreKAQ*—The questionnaire is self-administered, includes 9 items and investigates neglect-like symptoms, reduced proprioceptive acuity, and issues of body shape and size. Each item has a 5-point Likert scale (ranging from “never” = 0 to “always” = 4), with higher scores indicating greater levels of knee-specific disrupted body-perception [10].

*Knee injury and osteoarthritis outcome score* (*KOOS)*—This is a 42-item self-administered questionnaire made of five subscales, labeled: Pain, Symptoms, Activities of Daily Living (ADLs), Sport/Recreation (Sport/Rec), and knee-related Quality of Life (QoL).

In this study, the Sport/Rec subscale was not administered due to the characteristics of the population enrolled.

A 5-point Likert scale ranging from 0 (no problems) to 4 (extreme problems) is used to score each item, and the raw scores of each subscale are separately transformed into a 0–100 scale, with 0 indicating the worst problems and 100 indicating no problems [14].

*Pain intensity numerical rating scale* (PI-NRS)—An 11-point numerical rating scale ranging from 0 (no pain at all) to 10 (the worst imaginable pain) was used [15]. Participants were asked to rate their current pain intensity at both rest and during movement.

*Pain catastrophising scale (PCS)*—This is a 13-item self-report questionnaire in which participants are asked to rate the degree to which they have any of the thoughts described in the questionnaire using a five-point scale, ranging from 0 (never) to 4 (always). The total score is calculated by adding the scores of the individual items, and ranges from 0 to 52 [16].

*Hospital anxiety and depression score (HADS)*—The scale is composed of 14 items that create subscale scores for anxiety (HADS-A, 7 items) and depression (HADS-D, 7 items). The total score for each subscale is calculated by adding the scores of the individual items (0–3) and varies from 0 to 21 [17].

Other than the FreKAQ, all of the above measures previously demonstrated satisfactory psychometric properties in Italian populations [14–18].

### Cross-cultural adaptation of the FreKAQ

Adaptation of the FreKAQ was done in accordance with the protocol issued by the American Association of Orthopaedic Surgeon Outcomes Committee. The principles of good practice for the translation and cultural adaptation process for patient-reported outcomes measures based on the report of the International Society for Pharmacoeconomics and Outcomes Research (ISPOR) Task Force were also taken into account [19, 20].

*Step 1 Translation into Italian.* The items taken from the English version of the original FreKAQ tool [10] were translated into Italian with the aim of retaining the concepts of the original while using culturally and clinically fitting expressions. Two adaptations were made independently by 2 Italian professional translators experienced in the biomedical field. The translators were given a clear explanation of the concepts expressed to capture the conceptual meaning of the items, while keeping the language colloquial and compatible with a reading age of 12 years. Discrepancies between the translators were resolved by consensus. Step 1 ended when a common adaptation was agreed.

*Step 2 Back-Translation into English.* Two bilingual English native speaking translators, without any biomedical background, independently back-translated the initial adaptation. The principle investigator reviewed these translations and, with the help of the back-translators, made sure the Italian version reflected the same item content as the original version and was conceptually equivalent.

*Step 3 Expert committee.* To achieve harmonization of the adaptation process, the adaptations were submitted to a bilingual committee of clinicians, methodologists, and translators, chaired by the principle investigator. To identify any discrepancies or mistakes, the committee explored the semantic, idiomatic, and conceptual equivalence of the items and response options. This phase ended when a prefinal version was agreed.

*Step 4 Test of the prefinal version.* A test of the prefinal version was performed to investigate the level of acceptability, comprehensibility, interpretation and relevance of the adaptation, to highlight any items that may be inadequate at a conceptual level and to identify any other issues that cause uncertainty. Cognitive interviews were conducted by a trained psychologist who administered the new measure to 10 subjects with primary knee OA. These interviews were semi-structured and related to the following items: (1) was the questionnaire acceptable and easy to complete?; (2) how clear and comprehensible was the instruction to you?; (3) how clear and comprehensible were the questions to you?; (4) how clear and comprehensible were the response options to you?; (5) are there any questions which should be phrased in a clearer way?; (6) are there any questions which are too similar or irrelevant?

The Expert Committee reviewed the results from cognitive debriefing with the aim of identifying any change important for amelioration of the Italian prefinal version.

*Step 5 Submission to the developer*. The material related to the cross-cultural adaptation of the questionnaire was sent to the scale developer (B.M.W) for ultimate review. The final version is available in Additional file 1.

### Statistical analysis

The following items were analyzed.

#### Acceptability

The time needed to answer the questionnaire was recorded. The subjects were asked about any problems they encountered and the data were checked for missing or multiple responses.

#### Descriptive statistics

Descriptive statistics of collected measures was calculated, including indices of central tendency (mean) and spread (standard deviation), as well as percentage of minimum and maximum scores (floor or ceiling effect was considered to be present if > 15% of the subjects achieved the lowest or highest possible scores, respectively) [21].

#### Construct validity

1. *Internal construct (structural) validity*. Rasch analysis (Winsteps software v. 3.68.3) was used to examine the main psychometric properties of the FreKAQ-I with the rating scale model (due to the common response structure). We followed the same procedure detailed in our previous studies [22, 23]. In short, to investigate whether the pattern of responses met the assumptions of the Rasch model, the following psychometric issues were examined:
  - Functioning of the response categories, according to criteria suggested by Linacre [24]
  - Internal construct validity, calculated from each item’s chi-square fit statistics (infit and outfit mean-square statistics, MnSq). Values from 0.70 to 1.30 [25, 26], associated with standardized *z* values (ZStd) less than 2.0, were considered as an indicator of acceptable fit
  - Reliability, in terms of person and item reliability, providing the degree of replicability of person and item placements along the trait continuum [27]
  - Unidimensionality of the scale, examining the unexplained variance after the Rasch dimension is extracted, as obtained by a Principal Component Analysis of the residuals (PCAr). Additional factors are not likely to be present in the residuals if at least 50% of the variance is explained by the Rasch factor, and the eigenvalue of the first residual factor is ≤ 2 [24]
  - Local independence between items. No residual association among item responses should be found once the dominant factor (Rasch factor) has been conditioned out; a correlation of residuals > 0.30 indicates possible local dependence [28]
2. *External construct validity—*Based on previous studies describing the development of the Fremantle body awareness questionnaires and the maladaptive perceptions model proposed by Wand et al. [29], and Nishigami et al. [10], we examined external construct validity of the Italian version of the FreKAQ in the following way.
  - As for hypotheses testing, it was hypothesized a priori—that the FreKAQ score would achieve positive significant correlations (at fair to moderate levels, between 0.30 and 0.60) with measures analyzing other constructs pertinent to individuals with knee osteoarthritis, such as the KOOS subscales (symptoms, pain, ADL function and QoL), pain intensity (PI-NRS), pain catastrophizing (PCS), and anxiety/depression levels (HADS). After plot inspection for linearity of associations, correlations were calculated with Spearman’s rank correlation coefficient.
  - The known-group method was applied to evaluate the ability of the FreKAQ scores to discriminate between two groups of participants with different clinical profiles. We assessed: the four KOOS subscales (symptoms, pain, ADL function, and QoL) and pain intensity (dichotomized by group median); pain catastrophizing (< 24 points vs. ≥ 24 points) [30]; anxiety and depression levels (< 8 points vs. ≥ 8 points in each subscale) [31]. Differences between groups were assessed using the Mann–Whitney U test with Bonferroni correction for multiple comparisons with alpha set at 0.0056 (0.05/9).

Construct validity was considered good if ≥ 75% of the hypotheses (14 out of 18) was met.

#### Reliability and measurement error

Internal consistency was investigated through Cronbach’s alpha (values of > 0.70 being considered acceptable). Test–retest reliability (intraclass correlation coefficient, ICC_2.1_, with good and excellent reliability respectively indicated by values of 0.70–0.85 and > 0.85) [21] was examined. The SEM was estimated using the formula:

$$SEM = SD\sqrt {1 - ICC_{2,1} }$$

where SD is the baseline standard deviation of the measurements [21].

The minimum detectable change (MDC) was calculated by the equation:

$$MDC = SEM*z\,value*\sqrt 2$$

The 95% confidence level (CI) (MDC_95_) corresponds to a z value of 1.96.

Analyses were performed with Winsteps v. 3.68.2 (Winsteps.com, Beaverton—OR, USA) [24] for Rasch analysis and with R and RStudio [32, 33] for the other analyses.

We determined that a sample size of 101 would provide adequate statistical power (expecting to obtain an ICC of 0.70, with a 95%CI of 0.20) for test–retest reliability [34]. Moreover, in Rasch analysis a sample size of 100 participants is able to ensure item calibration stability within ± 0.5 logits with 95% confidence.

## Results

### Subjects

One hundred-thirty subjects were invited to participate, of whom 20 were excluded. The reasons for exclusion were, systemic illness (n = 3), cognitive impairment (n = 3), recent myocardial infarction (n = 2), recent cerebrovascular event (n = 1) and unwillingness to participate (n = 11). Of the remaining 110 subjects, 8 dropped out before starting the study because of, logistic problems (n = 5), economic constraints (n = 1), or personal problems (n = 2). Thus, the final study population consisted of 102 subjects, whose socio-demographic and clinical characteristics are reported in Table 1.

**Table 1:** Socio-demographic and clinical characteristics of the study population (n = 102)

Age (years), mean ± SD	69.1 ± 9.0
Pain duration (years), mean ± SD	1.4 ± 3.5
Body mass index (kg/m^2^), mean ± SD	28 ± 4
*Sex*	
Male	36
Female	66
*Married*	
Yes	86
No	16
*Employment*	
Not working due to pain	7
Employed	15
Self-employed	10
Retired	65
Unemployed	5
*Education level*	
Primary school	22
Middle school	30
High school	39
University	11
*Smokers*	
Yes	12
No	90

SD = standard deviation

### Translation and cross-cultural adaptation

The translation procedure took 1 month to reach a culturally adapted version, and all the items were easily forward and back-translated. No difficulties were evidenced during the review of the back translations. Some concern was raised related to the difficulty in discriminating between the Italian terms “rarely” versus “occasionally”, and “some of the time” versus “moderate amount of time”. Accordingly, the expert committee decided to simplify the labels of the rating categories maintaining just 5 simple descriptors: “never”, “rarely”, “sometimes”, “often”, “always”. The correctness of the process, the content of the items and the concepts expressed were confirmed by the experts. Then, the research panel reviewed the results from cognitive interviews and made only small adjustments based on interpretations and meanings raised by the subjects in order to improve items comprehension. As no other significant issues were pointed out further, the principal investigator and the Expert committees confirmed the work done and finalized the definitive version of the translation.

### Scale psychometric properties

#### Acceptability

All of the questions were well accepted. The questionnaire was completed in about 4 min. No missing responses or multiple answers were found. There were no further problems in comprehension.

#### Descriptive statistics

Mean and standard deviation of the scores of each collected measure, as well as percentage of minimum and maximum scores are reported in Table 2. No floor or ceiling effect was observed in any of the questionnaires used, including the FreKAQ-I.

**Table 2:** Distributions (mean and standard deviation, SD) and percentage of minimum and maximum scores of the scores of the Fremantle Knee Awareness Questionnaire (FreKAQ), Knee injury and osteoarthritis outcome score (KOOS), pain intensity numerical rating scale (PI-NRS), pain catastrophising scale (PCS), and Hospital anxiety and depression score (HADS)

Measures (range)	Mean (SD)	Minimum scores (%)	Maximum scores (%)
FreKAQ (0–36)	14 (7)	3.9	2.0
KOOS Pain (0–100)	51.4 (17.1)	1.0	1.0
KOOS Symptoms (0–100)	48.9 (21.5)	1.0	2.9
KOOS ADLs (0–100)	52.4 (19.8)	2.9	2.0
KOOS QoL (0–100)	76.2 (15.1)	1.0	7.8
PI-NRS rest (0–10)	3.8 (2.3)	3.9	2.9
PI-NRS motion (0–10)	7.4 (2.1)	1.0	13.7
PCS (13–52)	23.1 (11.8)	2.0	1.0
HADS Anxiety (0–21)	6.8 (3.3)	2.9	1.0
HADS Depression (0–21)	5.5 (3.3)	3.9	1.0

*ADLs* activities of daily living, *QoL* quality of life

#### Construct validity

*Internal construct (structural) validity*. Rasch analysis showed that in the 5-level rating scale the average category measures advanced monotonically and the category oufit mean-square were less than 2.0. However, some step calibrations were disordered, and the differences between step difficulties were quite narrow (always < 0.98 logits).

All items fitted the underlying construct that the scale was intended to measure (Table 3). The mean person ability was − 0.35 logits (range from − 3.29 to 0.85), indicating that the average difficulty of the items (endorsability) was quite well targeted to the mean sample ability (agreeability). The item reliability was 0.98, while person reliability was 0.67. The PCAr confirmed the substantial unidimensionality of the FreKAQ: the variance explained by the Rasch factor was 53.3%, while the unexplained variance in the first contrast showed an eigenvalue of 1.8. No local dependence was detected, i.e. no strong (> 0.0.20) residual correlation between items was found.

**Table 3:** Summary of Rasch analysis results related to the FreKAQ, containing item-difficulty measures (with standard error, SE) and fit information

Item number and label	Measure (SE)	Infit		Outfit	
		MnSq	ZStd	MnSq	ZStd
1. My sore knee feels as though it is not part of the rest of my body	0.60 (0,10)	0.89	− 0.7	1.01	0.1
2. I need to focus all my attention on my sore knee to make it move the way I want it to	− 0.09 (0.08)	0.78	− 1.9	0.74	− 1.8
3. I feel as if my sore knee sometimes moves involuntarily, without my control	0.49 (0.09)	0.75	− 1.8	1.22	1.0
4. When performing everyday tasks, I don’t know how much my sore knee is moving	0.57 (0.10)	0.96	− 0.2	1.29	1.2
5. When performing everyday tasks, I am not sure exactly what position my sore knee is in	0.75 (0.11)	1.05	0.4	0.96	− 0.1
6. When my knee is hidden from view, I can’t perceive the exact outline of my sore knee	0.13 (0.09)	1.20	1.5	1.29	1.8
7. My sore knee feels larger than it appears	− 0.70 (0.09)	0.91	− 0.6	1.19	1.1
8. My sore knee feels smaller than it appears	− 0.85 (0.09)	1.11	0.8	1.11	0.7
9. My knees feel different between left and right (in terms of size and shape)	− 0.91 (0.09)	1.11	0.8	1.24	1.8

Item difficulty is expressed in logit units, a logit being the natural logarithm of the ratio (odds) of mutually exclusive alternatives (e.g. higher response vs. lower response): zero logits represents mean item difficulty, and negative measures indicate higher frequency of occurrence with that statement (i.e. higher scores in that item)

MnSq = mean-square statistics; ZStd = standardized *z* value

#### External construct validity

External construct validity was considered good as:

1. All a priori hypotheses were confirmed. Related results can be seen in Table 4.
2. As for the known-group method, FreKAQ scores were significantly different between two groups of participants with different levels (high vs. low) of the following clinical variables: the four KOOS subscales (symptoms, pain, ADL function, and QoL); pain intensity; pain catastrophizing; anxiety and depression levels (for all variables, *p* ≤ 0.004).

**Table 4:** Construct validity. Spearman’s correlations between the FreKAQ-I and KOOS, PI-NRS, PCS and HADS. Confidence intervals by bootstrap, 1000 repetitions

Measures versus FreKAQ-I	Spearman’s correlation		
	rho	95% Conf. Interval	
KOOS pain	0.51*	0.35	0.66
KOOS symptoms	0.50*	0.34	0.65
KOOS ADLs	0.59*	0.44	0.73
KOOS QoL	0.38*	0.20	0.56
PI-NRS rest	0.35*	0.16	0.54
PI-NRS motion	0.37*	0.19	0.54
PCS	0.47*	0.32	0.63
HADS anxiety	0.36*	0.18	0.54
HADS depression	0.43*	0.28	0.58

*p < .001

*FreKAQ-I* Fremantle knee awareness questionnaire, Italian version, *KOOS* Knee injury and osteoarthritis outcome score, *ADLs* activities of daily living, *QoL* quality of life, *PI-NRS* pain intensity numerical rating scale, *PCS* pain catastrophising scale, *HADS* Hospital anxiety and depression score

#### Reliability and measurement error

Cronbach’s α was 0.74. Test–retest reliability was excellent (ICC = 0.92; CI 0.87–0.94). The SEM was 1.88 and the MDC_95_ was 5.22, reflecting the smallest change in score that is likely to reflect a true change rather than a measurement error.

## Discussion

This study describes the process of cross-cultural adaptation of the FreKAQ and evaluation of structural validity, reliability, measurement error and construct validity of the questionnaire in Italian-speaking subjects with painful knee OA. The scale confirmed its unidimensionality, displayed good construct validity and acceptable reliability.

Cross-cultural adaptation requires a process of translation, backward translation, expert committee revision and testing of the pre-final version in order to guarantee the meaning of the original items are adequately captured in the Italian language. These recommended guidelines were followed in the current study and all of the steps indicated that cross cultural adaptation of the FreKAQ was successful. The on-field test with cognitive debriefing confirmed the overall general comprehensibility of the translated questionnaire. The decision to simplify the labels of the rating categories maintaining just 5 simple descriptors: “never”, “rarely”, “sometimes”, “often”, “always” is in line with other widely-used scales, such as some of those in the PROMIS item bank [35].

The final version of the questionnaire was highly acceptable, easily understood and capable of being self-administered. The questionnaires burden is also low as it required less than five minutes to complete. It therefore seems to be applicable in everyday clinical practice. However, among various content validity aspects, only the comprehensibility of the FreKAQ was assessed in this study, whereas its relevance and comprehensiveness to measure body perception was not assessed; this research gap should be filled by future studies conducting a thorough assessment of content validity, possibly following the recently published COSMIN methodology [36].

### Construct validity

#### Internal construct (structural) validity

Overall, Rasch analysis of the FreKAQ-I showed that the scale has acceptable psychometric properties. The scale’s unidimensionality was confirmed, all items fit the Rasch model, and no local item dependence was found. In addition, the targeting of item difficulty to patient ability was quite good, and item reliability was high.

The only concern came from the rating scale diagnostics, which showed some category misfunctioning due to the limited respondents’ ability to discern between the three central levels of frequency. This finding is in agreement with observations indicating problems with the use of the 5-grade frequency-related response categories, often called ‘vague quantifiers’; there is neither a formal nor an informal definition given for the meaning of terms such as ‘sometimes’, ‘rarely’, ‘occasionally’, and thus they have fuzzy boundaries [37]. Furthermore, the intervals between category steps were quite narrow, in line with those in the original paper [10] where they were lower than 0.8 logits.

The FreKAQ is based on a formative model, i.e., the latent construct (here “knee awareness”) is determined as a combination of independent—albeit correlated—indicators of the variable under measurement, thus creating a composite index. Items are a mix of positively- and negatively-worded questions respectively related to neglect-like symptoms (items 1–3), reduced proprioceptive acuity (items 4 and 5), and perceived body shape and size (items 7–9), with item 6 alternatively assigned to the second or the third domain.

As such, these items do not necessarily have good correlations or orient in a consistent hierarchy.

#### External construct validity

As for hypotheses testing, the correlation with a measure of disability (KOOS) was as expected (Table 3), suggesting patients showing high disability due to painful knee OA also display greater levels of body perception disruption specific to the knee. This is in keeping with results reported for the Japanese version of the questionnaire [10], where a moderate correlation with disability was also noted (0.41). However, these authors used a different measure of disability (i.e., The Oxford Knee Score) so a detailed comparison with the current findings cannot be undertaken. As for correlation with pain intensity, estimates were also as expected, with higher levels of pain being associated with higher levels of perceptual dysfunction, in line with results reported for the Japanese version of the questionnaire [10]. Both the Japanese study and the current study examined knee pain intensity at rest and with motion and report remarkably consistent findings. Results from both the Japanese [10] and current study suggest that disrupted body perception is more strongly related to disability than pain in people with painful knee OA. This trend is also seen in various studies of the lumbar spine version of the questionnaire in people with back pain [29, 38–40]. Together these results suggest that disrupted body perception may impact more on the functional consequences of pain rather than the experience of pain itself. Future investigations that examine the role of body perception in mediating the relationship between pain and disability may be worth exploring.

With respect to pain catastrophising, we achieved a lower correlation than reported for the Japanese version of the FreKAQ (0.70). It is possible that in the Italian clinical context some FreKAQ and PCS items are interpreted differently than in Japan, hence leading to a different correlation than previously found. As for mood disorders, our correlations substantially replicate the moderate correlations already achieved in the original study (rho = 0.36 and 0.43 with anxiety and depression scores, respectively) [10], suggesting that patients suffering from anxiety and depression are more prone to show disrupted body perception specific to the knee.

Moreover, data related to known-group validity supported previous conceptualizations and reports indicating that a measure of impaired body perception (FreKAQ scores) would be able to discriminate between participants with different levels of knee symptoms, function, pain intensity, pain catastrophizing and psychological distress [10, 29, 41].

### Reliability and measurement error

The internal consistency of the FreKAQ-I was acceptable (0.72), but inferior to that reported in the original study (0.88) [10]. However, this low internal consistency is unsurprising, when taking into account both the formative model of the scale, and similar results seen from another modified version of the Fremantle Awareness Questionnaires examining self-perceptions in neck disorders [42]. This result, coupled with the low Rasch person reliability, indicates that the tool is useful for group-level comparisons but less so for clinical application in single individuals (for whom a minimum reliability threshold of 0.85–0.90 is desirable). These results suggest the scale seems able to clearly distinguish between persons with impaired versus not-impaired knee self-perception without permitting a more fine grained assessment of impairment levels.

Test–retest reliability showed an excellent agreement between the results on days 1 and 8, even higher than reported in the original scale [10]. The FreKAQ-I displayed an acceptable measurement error. Given the high degree of repeatability of our results, the SEM and MDC were relatively small: in particular, the MDC indicated—at a 95% confidence level—that, if an individual shows a change of more than 6 points after a given intervention, it would not be due to a measurement error.

This study has some limitations. Firstly, it is a cross-sectional study and responsiveness as well as minimal important change of the FreKAQ-I could not be assessed. Secondly, the relationships between knee-related perceptual dysfunction and physical performance measures were not considered because only questionnaires were used. Thirdly, correlations with other measures including psychological factors (e.g. Tampa Scale of Kinesiophobia or Pain Self-Efficacy Questionnaire) and quality of life issues (e.g. Short-Form Health Survey 36-items) available in Italian language were not analyzed [43–45]. Fourthly, our study was restricted to patients with primary knee OA and it is uncertain whether these findings can be extended to patients with other causes of knee pain, and future studies in these populations are recommended.

## Conclusions

The Italian version of the FreKAQ shows a one-factor structure, it is reliable and valid, and has an acceptable measurement error. The newly adapted tool can be recommended for clinical and research purposes in order to improve the assessment and treatment planning of patients with painful knee OA in Italy.

## Supplementary Information

**Additional file 1.** The Italian version of the Fremantle Knee Awareness Questionnaire.

## Data Availability

The datasets used and/or analysed during the current study are available from the corresponding author on reasonable request.

## Abbreviations

CI: Confidence interval
FreKAQ: Fremantle knee awareness questionnaire
FreKAQ-I: Fremantle knee awareness questionnaire-Italian
HADS: Hospital anxiety and depression score
HADS-A: Hospital anxiety and depression score-anxiety
HADS-D: Hospital anxiety and depression score-depression
ICC: Intraclass correlation coefficient
ISPOR: International Society for Pharmacoeconomics and Outcomes Research
KOOS: Knee injury and osteoarthritis outcome score
MDC: Minimum detectable change
MnSq: Mean-square statistics
OA: Osteoarthritis
PCAr: Principal component analysis of the residuals
PCS: Pain catastrophising scale
PI-NRS: Pain intensity numerical rating scale
SD: Standard error
SEM: Standard error of the mean
ZStd: Standardized *z* values
