# Supplementary material for: Cross-cultural adaptation, reliability and validity of the Fremantle Knee Awareness Questionnaire in Italian subjects with painful knee osteoarthritis
Source: Health Qual Life Outcomes. 2021 Apr 7;19:114. doi: 10.1186/s12955-021-01754-4 (PMC8025485; doi:10.1186/s12955-021-01754-4)
Supplement: Supplementary file 1 — Additional file 1. The Italian version of the Fremantle Knee Awareness Questionnaire. [file 12955_2021_1754_MOESM1_ESM.doc]

**Fremantle Knee Awareness Questionnaire – Italian version**

Di seguito sono descritte alcune affermazioni che altre persone ci hanno riportato su come sentono il loro ginocchio dolorante. Usando la scala seguente, per cortesia indichi come percepisce il suo ginocchio quando avverte dolore. Se avverte dolore in entrambe le ginocchia, risponda alla domanda facendo riferimento al ginocchio più dolorante.

0 = Mai percepito così

1 = Percepito così raramente

2 = Percepito così occasionalmente

3 = Percepito spesso così

4 = Percepito sempre così

|  | Mai | Raramente | Qualche volta | Spesso | Sempre |
| --- | --- | --- | --- | --- | --- |
| 1. Sembra che il ginocchio dolorante non faccia parte del resto del mio corpo |  |  |  |  |  |
| 2. Deve concentrarmi moltissimo per muovere il ginocchio dolorante come voglio |  |  |  |  |  |
| 3. Qualche volta mi sembra che il ginocchio dolorante si muova indipendentemente dalla mia volontà |  |  |  |  |  |
| 4. Non mi rendo conto dei movimenti del mio ginocchio dolorante quando svolgo le attività quotidiane (a casa, al lavoro, etc.) |  |  |  |  |  |
| 5. Non sono sicuro in che posizione è esattamente il mio ginocchio dolorante quando svolgo le attività quotidiane (a casa, al lavoro, etc.) |  |  |  |  |  |
| 6. Non riesco ad immaginare in modo corretto come è fatto il mio ginocchio dolorante quando è nascosto alla vista |  |  |  |  |  |
| 7. Mi sembra che il ginocchio dolorante sia più grande di quel che appare |  |  |  |  |  |
| 8. Mi sembra che il ginocchio dolorante sia più piccolo di quel che appare |  |  |  |  |  |
| 9. Mi sembra che il ginocchio destro sia diverso da quello sinistro (in termini di grandezza e forma) |  |  |  |  |  |

Punteggio: …../36
